# Supplementary material for: Phase shift in skyrmion crystals
Source: Nat Commun. 2021 Dec 1;12:6927. doi: 10.1038/s41467-021-27083-0 (PMC8636495; doi:10.1038/s41467-021-27083-0)
Supplement: Supplementary file 1 — Supplementary Information [file 41467_2021_27083_MOESM1_ESM.pdf]

Supplementary Information for "Phase Shift in Skyrmion Crystals"

Satoru Hayami[1], Tsuyoshi Okubo[2], and Yukitoshi Motome[1]
[1]*Department of Applied Physics, The University of Tokyo, Tokyo 113-8656, Japan*
[2]*Institute for Physics of Intelligence, The University of Tokyo, Tokyo 113-0033, Japan*

Supplementary Note 1. Tetra-axial vortex crystal

We describe the details of the spin configuration in the tetra-axial vortex crystal (TVX) introduced in the main text. The vortex cores are identified by calculating the vorticity $l^i_{\mu\nu}$ at ith spin in the $\mu\nu$ plane as

$$l^i_{\mu\nu} = \frac{1}{2\pi}\sum_j (\phi^{\mu\nu}_{i,j+1} - \phi^{\mu\nu}_{i,j}), \tag{1}$$

where $\phi^{\mu\nu}_{i,j}$ is the angle of jth spin measured from the μ-axis on the $\mu\nu$ plane; the index j runs the neighboring spins around the ith spin. $l^i_{\mu\nu}$ takes an integer for nonzero vorticity. As shown in Supplementary Figs. 1a and 1b, there are four types of vortices in the magnetic unit cell, which possess different sets of (l_{yz}, l_{zx}, l_{xy}) around the different cores: $(l_{yz}, l_{zx}, l_{xy}) = (+1, +1, +1)$ at the cores denoted by the blue dots, $(+1, -1, -1)$ at the red dots, $(-1, +1, -1)$ at the green dots, and $(-1, -1, +1)$ at the black dots. Thus, the four types of vortices comprise a tetrahedron in the parameter space of (l_{yz}, l_{zx}, l_{xy}), as shown in Supplementary Fig. 1c. This is why we call the spin texture the TVX in the main text.

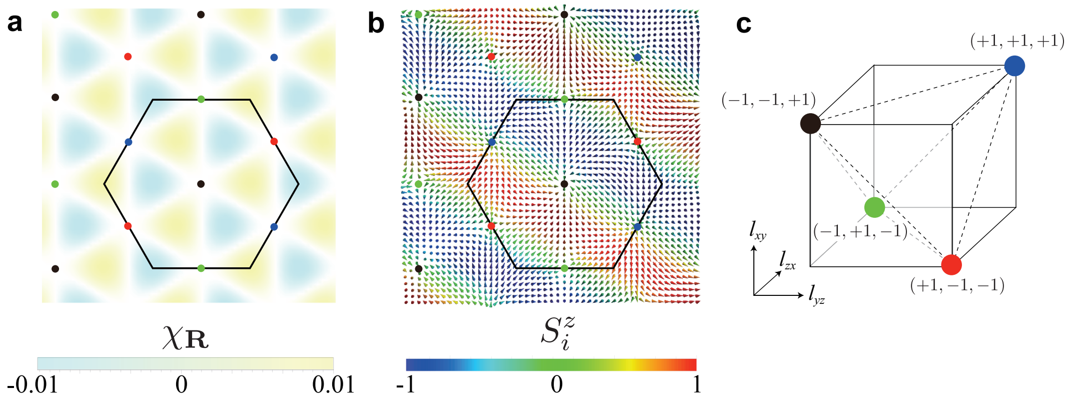

Supplementary Fig. 1: **a**, Scalar spin chirality $\chi_{\mathbf{R}}$ and **b**, spin configurations in the tetra-axial vortex crystal (TVX) when taking $\theta_1 = \pi/3$, $\theta_2 = \pi/6$ and $\theta_3 = 0$ for $\mathbf{S}^{\rm sin}_i$ in the main text. The solid hexagons in **a** and **b** represent the magnetic unit cell. **c**, The positions of the vortex cores are denoted by the dots in **a** and **b** whose colors represent the different sets of vorticity, (l_{yz}, l_{zx}, l_{xy}).

As mentioned in the main text, the different spin textures are obtained while changing the set of phases, $(\theta_1, \theta_2, \theta_3)$, on a discrete lattice. The optimal set of the phases are chosen so that the grand potential Ω becomes minimum. We show the contour plot of Ω while changing θ_1 and θ_2 for the TVX with $\Theta = \pi/2$, the $n_{\rm sk} = 2$ skyrmion crystal (SkX2) with $\Theta = 0$, and the $n_{\rm sk} = 1$ skyrmion crystal (SkX1) with $\Theta = 0$ in Supplementary Fig. 2 in the Kondo lattice model with $t_1 = 1$, $t_3 = -0.85$, $J_{\rm K} = 1$, and $\mu = -3.5$ giving $Q = \pi/3$. Note that in all the cases the variational states are prepared for given θ_1 and θ_2 with the normalisation of the spin length at each site. The grand potential in each 3Q state takes minima at symmetry-related $(\theta_1, \theta_2, \theta_3)$, which are denoted as the green circles in Supplementary Fig. 2. We find that the set of phases are chosen by commensurability of the spin textures to the underlying lattice: the skyrmion and vortex cores are located at the interstitial sites instead of the lattice sites for the optimal phases. We show real-space spin textures in the TVX with $(\theta_1, \theta_2, \theta_3) = (\pi/3, \pi/6, 0)$ in Supplementary Fig. 3a and $(0, 0, \pi/2)$ in Supplementary Fig. 3b with $Q = \pi/3$ $(\pi/6)$. We note that the spin texture with $(\theta_1, \theta_2, \theta_3) = (\pi/3, \pi/6, 0)$ at $Q = \pi/3$ in Supplementary Fig. 3a gives the lowest variational energy of the Kondo lattice model and the effective spin model in the main text (see also Supplementary Fig. 2a). We also show real-space spin textures in the $n_{\rm sk} = 2$ SkX2 with $(\theta_1, \theta_2, \theta_3) = (0, 0, 0)$ in Supplementary Fig. 3c and $(\pi/3, -\pi/3, 0)$ in Supplementary Fig. 3d with $Q = \pi/3$. The optimized spin texture at $Q = \pi/3$ by the variational calculations in the main text corresponds to that in Supplementary Fig. 3d (see also Supplementary Fig. 2b).

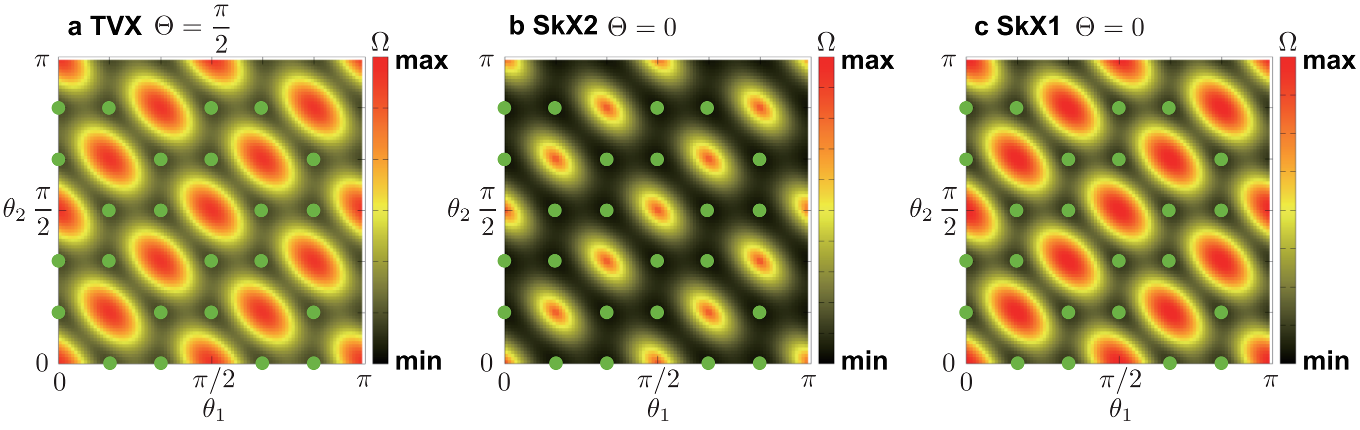

Supplementary Fig. 2: **a**-**c**, Grand potential Ω in the plane of θ_1 and θ_2 with $Q = \pi/3$: the TVX with $\Theta = \pi/2$ (**a**), the SkX2 with $\Theta = 0$ (**b**), and the SkX1 with $\Theta = 0$ (**c**). The optimal phases giving the lowest Ω are denoted as the green circles.

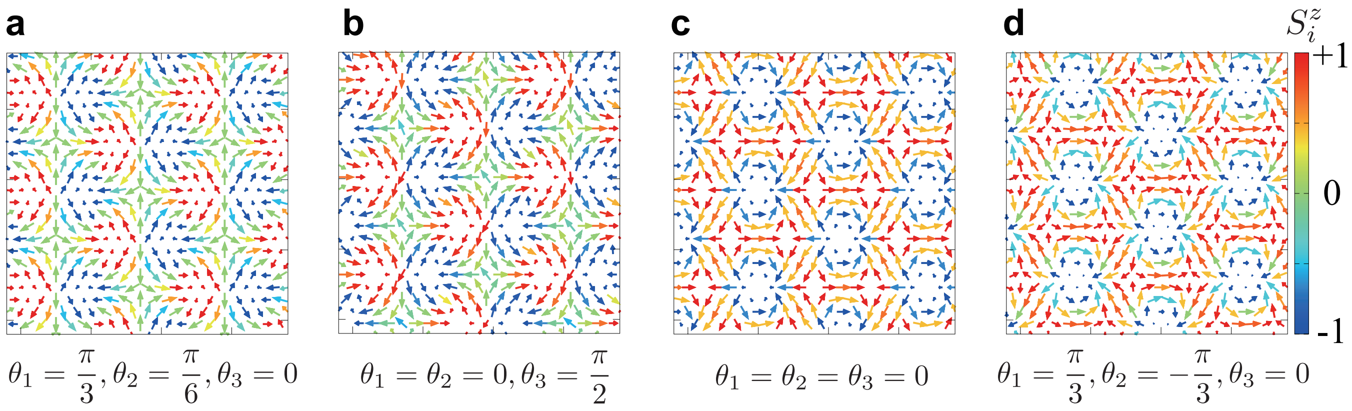

Supplementary Fig. 3: **a**-**d**, Spin configurations of the triple sinusoidal crystals: the TVX (**a**,**b**) and the SkX2 (**c**,**d**) with $Q = \pi/3$. The phases $(\theta_1, \theta_2, \theta_3)$ are taken at $(\pi/3, \pi/6, 0)$ (**a**), $(0, 0, \pi/2)$ (**b**), $(0, 0, 0)$ (**c**), and $(\pi/3, -\pi/3, 0)$ (**d**).

Supplementary Note 2. Real-space spin textures in the anisotropic triple-Q state for the Kondo lattice model

We present the real-space spin configuration in the anisotropic triple-Q state obtained for the Kondo lattice model by the Langevin dynamics simulations with the kernel polynomial method. Supplementary Fig. 4 represents a snapshot in the anisotropic triple-Q state at $T = 0.006$ and $H = 0.004$. As clearly shown in Supplementary Fig. 4, the vortex cores in the anisotropic triple-Q state are located at the nodes where the sign of the spin component perpendicular to the vortex plane is reversed. This tendency is similar to that in the TVX state stabilized at $H = 0$.

Supplementary Note 3. Magnetic chirality interaction

We show that the effective magnetic chirality interaction with the coupling constant L in the model in equation (4) in Methods is naturally obtained by the perturbation theory for the Kondo lattice model in equation (1) without the magnetic field. By considering the situation where the exchange coupling J_{K} is small enough compared to the bandwidth of itinerant electrons, the free energy can be expanded in terms of $2n$th order of J_{K}. As discussed in Supplementary Ref. [1], the important contributions of the lowest and second lowest order are described by the first and second terms in equation (4). They do not affect the phase in the SkXs, as they consist of the spins with equal number of $+\mathbf{Q}_\nu$ and $-\mathbf{Q}_\nu$, compatible with the translational symmetry.

The lowest-order interaction relevant to the phase is obtained from the sixth-order process in terms of J_{K} under the constraint $\mathbf{Q}_1 + \mathbf{Q}_2 + \mathbf{Q}_3 = \mathbf{0}$ in the present triangular-lattice system. In general, the sixth-order contribution to the free energy is described

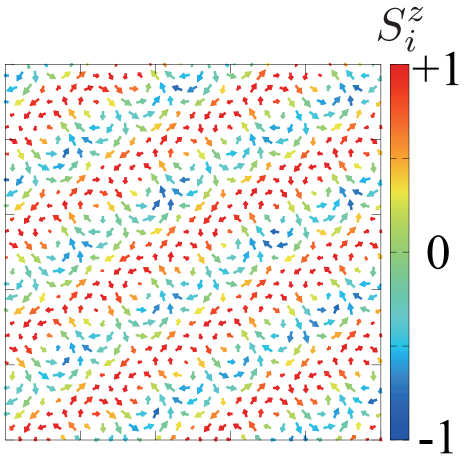

Supplementary Fig. 4: Real-space spin configurations in the anisotropic triple-Q ($3Q$) state obtained by the Langevin dynamics simulations with the kernel polynomial method for the Kondo lattice model with $N = 96^2$ at $T = 0.006$ and $H = 0.004$.

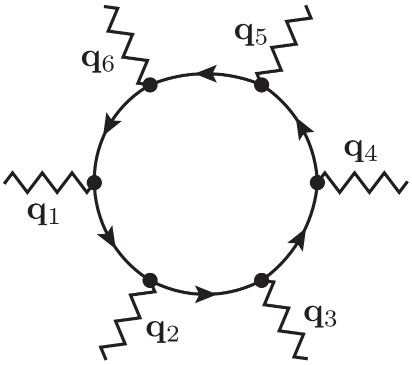

Supplementary Fig. 5: Feynman diagram for the sixth-order terms in the perturbative expansion of the free energy in the Kondo lattice model. The vertices with wavy lines denote the scattering by localized spins and the solid curves represent the bare propagators of itinerant electrons, $G_{\mathbf{k}}$.

by

$$
\begin{aligned}
F^{(6)} =& \frac{T}{3}\frac{J_{\rm K}^6}{N^3} \sum_{\mathbf{k},\omega_p} \sum_{\mathbf{q}_1,\mathbf{q}_2,\mathbf{q}_3,\mathbf{q}_4,\mathbf{q}_5,\mathbf{q}_6} G_{\mathbf{k}} G_{\mathbf{k}+\mathbf{q}_1} G_{\mathbf{k}+\mathbf{q}_1+\mathbf{q}_2} G_{\mathbf{k}+\mathbf{q}_1+\mathbf{q}_2+\mathbf{q}_3} G_{\mathbf{k}+\mathbf{q}_1+\mathbf{q}_2+\mathbf{q}_3+\mathbf{q}_4} G_{\mathbf{k}+\mathbf{q}_1+\mathbf{q}_2+\mathbf{q}_3+\mathbf{q}_4+\mathbf{q}_5} \\
&\times [(\mathbf{S}_{\mathbf{q}_1}\cdot\mathbf{S}_{\mathbf{q}_2})(\mathbf{S}_{\mathbf{q}_3}\cdot\mathbf{S}_{\mathbf{q}_4})(\mathbf{S}_{\mathbf{q}_5}\cdot\mathbf{S}_{\mathbf{q}_6}) - (\mathbf{S}_{\mathbf{q}_1}\cdot\mathbf{S}_{\mathbf{q}_2})(\mathbf{S}_{\mathbf{q}_3}\cdot\mathbf{S}_{\mathbf{q}_5})(\mathbf{S}_{\mathbf{q}_4}\cdot\mathbf{S}_{\mathbf{q}_6}) + (\mathbf{S}_{\mathbf{q}_1}\cdot\mathbf{S}_{\mathbf{q}_2})(\mathbf{S}_{\mathbf{q}_3}\cdot\mathbf{S}_{\mathbf{q}_6})(\mathbf{S}_{\mathbf{q}_4}\cdot\mathbf{S}_{\mathbf{q}_5}) \\
&\quad -(\mathbf{S}_{\mathbf{q}_1}\cdot\mathbf{S}_{\mathbf{q}_3})(\mathbf{S}_{\mathbf{q}_2}\cdot\mathbf{S}_{\mathbf{q}_4})(\mathbf{S}_{\mathbf{q}_5}\cdot\mathbf{S}_{\mathbf{q}_6}) + (\mathbf{S}_{\mathbf{q}_1}\cdot\mathbf{S}_{\mathbf{q}_3})(\mathbf{S}_{\mathbf{q}_2}\cdot\mathbf{S}_{\mathbf{q}_5})(\mathbf{S}_{\mathbf{q}_4}\cdot\mathbf{S}_{\mathbf{q}_6}) - (\mathbf{S}_{\mathbf{q}_1}\cdot\mathbf{S}_{\mathbf{q}_3})(\mathbf{S}_{\mathbf{q}_2}\cdot\mathbf{S}_{\mathbf{q}_6})(\mathbf{S}_{\mathbf{q}_4}\cdot\mathbf{S}_{\mathbf{q}_5}) \\
&\quad +(\mathbf{S}_{\mathbf{q}_1}\cdot\mathbf{S}_{\mathbf{q}_4})(\mathbf{S}_{\mathbf{q}_2}\cdot\mathbf{S}_{\mathbf{q}_3})(\mathbf{S}_{\mathbf{q}_5}\cdot\mathbf{S}_{\mathbf{q}_6}) - (\mathbf{S}_{\mathbf{q}_1}\cdot\mathbf{S}_{\mathbf{q}_4})(\mathbf{S}_{\mathbf{q}_2}\cdot\mathbf{S}_{\mathbf{q}_5})(\mathbf{S}_{\mathbf{q}_3}\cdot\mathbf{S}_{\mathbf{q}_6}) + (\mathbf{S}_{\mathbf{q}_1}\cdot\mathbf{S}_{\mathbf{q}_4})(\mathbf{S}_{\mathbf{q}_2}\cdot\mathbf{S}_{\mathbf{q}_6})(\mathbf{S}_{\mathbf{q}_3}\cdot\mathbf{S}_{\mathbf{q}_5}) \\
&\quad -(\mathbf{S}_{\mathbf{q}_1}\cdot\mathbf{S}_{\mathbf{q}_5})(\mathbf{S}_{\mathbf{q}_2}\cdot\mathbf{S}_{\mathbf{q}_3})(\mathbf{S}_{\mathbf{q}_4}\cdot\mathbf{S}_{\mathbf{q}_6}) + (\mathbf{S}_{\mathbf{q}_1}\cdot\mathbf{S}_{\mathbf{q}_5})(\mathbf{S}_{\mathbf{q}_2}\cdot\mathbf{S}_{\mathbf{q}_4})(\mathbf{S}_{\mathbf{q}_3}\cdot\mathbf{S}_{\mathbf{q}_6}) - (\mathbf{S}_{\mathbf{q}_1}\cdot\mathbf{S}_{\mathbf{q}_5})(\mathbf{S}_{\mathbf{q}_2}\cdot\mathbf{S}_{\mathbf{q}_6})(\mathbf{S}_{\mathbf{q}_3}\cdot\mathbf{S}_{\mathbf{q}_4}) \\
&\quad +(\mathbf{S}_{\mathbf{q}_1}\cdot\mathbf{S}_{\mathbf{q}_6})(\mathbf{S}_{\mathbf{q}_2}\cdot\mathbf{S}_{\mathbf{q}_3})(\mathbf{S}_{\mathbf{q}_4}\cdot\mathbf{S}_{\mathbf{q}_5}) - (\mathbf{S}_{\mathbf{q}_1}\cdot\mathbf{S}_{\mathbf{q}_6})(\mathbf{S}_{\mathbf{q}_2}\cdot\mathbf{S}_{\mathbf{q}_4})(\mathbf{S}_{\mathbf{q}_3}\cdot\mathbf{S}_{\mathbf{q}_5}) + (\mathbf{S}_{\mathbf{q}_1}\cdot\mathbf{S}_{\mathbf{q}_6})(\mathbf{S}_{\mathbf{q}_2}\cdot\mathbf{S}_{\mathbf{q}_5})(\mathbf{S}_{\mathbf{q}_3}\cdot\mathbf{S}_{\mathbf{q}_4})] \\
&\times \delta_{\mathbf{q}_1+\mathbf{q}_2+\mathbf{q}_3+\mathbf{q}_4+\mathbf{q}_5+\mathbf{q}_6,0}, \qquad (2)
\end{aligned}
$$

where T is the temperature, $G_{\mathbf{k}}(i\omega_p) = [i\omega_p - (\varepsilon_{\mathbf{k}} - \mu)]^{-1}$ is noninteracting Green's function, $\varepsilon_{\mathbf{k}}$ is the energy dispersion, ω_p is the Matsubara frequency, μ is the chemical potential, and δ is the Kronecker delta. The Feynman diagram of the sixth-order contribution is shown in Supplementary Fig. 5, which consists of the vertices representing the scattering by localized spins and the bare propagators of itinerant electrons, $G_{\mathbf{k}}$.

Among the contributions in Supplementary equation (2), the terms including only positive $(\mathbf{Q}_1, \mathbf{Q}_2, \mathbf{Q}_3)$ or negative $(-\mathbf{Q}_1, -\mathbf{Q}_2, -\mathbf{Q}_3)$ play an important role in the phase shift. For positive $(\mathbf{Q}_1, \mathbf{Q}_2, \mathbf{Q}_3)$, the effective interaction $F^{(6+)}$ is

given by

$$F^{(6+)} = \frac{J_{\rm K}^6}{N^2}\left[A_1(\mathbf{S}_{\mathbf{Q}_1}\cdot\mathbf{S}_{\mathbf{Q}_1})(\mathbf{S}_{\mathbf{Q}_2}\cdot\mathbf{S}_{\mathbf{Q}_2})(\mathbf{S}_{\mathbf{Q}_3}\cdot\mathbf{S}_{\mathbf{Q}_3}) + A_2(\mathbf{S}_{\mathbf{Q}_1}\cdot\mathbf{S}_{\mathbf{Q}_2})(\mathbf{S}_{\mathbf{Q}_2}\cdot\mathbf{S}_{\mathbf{Q}_3})(\mathbf{S}_{\mathbf{Q}_3}\cdot\mathbf{S}_{\mathbf{Q}_1}) \right.$$
$$\left. + A_3\left\{(\mathbf{S}_{\mathbf{Q}_1}\cdot\mathbf{S}_{\mathbf{Q}_1})(\mathbf{S}_{\mathbf{Q}_2}\cdot\mathbf{S}_{\mathbf{Q}_3})^2 + (\mathbf{S}_{\mathbf{Q}_2}\cdot\mathbf{S}_{\mathbf{Q}_2})(\mathbf{S}_{\mathbf{Q}_1}\cdot\mathbf{S}_{\mathbf{Q}_3})^2 + (\mathbf{S}_{\mathbf{Q}_3}\cdot\mathbf{S}_{\mathbf{Q}_3})(\mathbf{S}_{\mathbf{Q}_1}\cdot\mathbf{S}_{\mathbf{Q}_2})^2\right\}\right], \tag{3}$$

where

$$A_1 = \frac{2T}{N}\sum_{\mathbf{k},\omega_p}\left[3(G_{\mathbf{k}})^2(G_{\mathbf{k}-\mathbf{Q}_1})^2(G_{\mathbf{k}+\mathbf{Q}_1})^2 + 3(G_{\mathbf{k}})^2 G_{\mathbf{k}-\mathbf{Q}_1}G_{\mathbf{k}+\mathbf{Q}_1}G_{\mathbf{k}-\mathbf{Q}_2}G_{\mathbf{k}+\mathbf{Q}_2} + 3(G_{\mathbf{k}})^2(G_{\mathbf{k}-\mathbf{Q}_1})^2 G_{\mathbf{k}+\mathbf{Q}_2}G_{\mathbf{k}+\mathbf{Q}_3}\right.$$
$$\left. -6(G_{\mathbf{k}})^2 G_{\mathbf{k}-\mathbf{Q}_1}G_{\mathbf{k}+\mathbf{Q}_1}G_{\mathbf{k}+\mathbf{Q}_2}G_{\mathbf{k}-\mathbf{Q}_3} + 2G_{\mathbf{k}}G_{\mathbf{k}-\mathbf{Q}_1}G_{\mathbf{k}+\mathbf{Q}_2}G_{\mathbf{k}-\mathbf{Q}_3}G_{\mathbf{k}+\mathbf{Q}_3}G_{\mathbf{k}-\mathbf{Q}_1+\mathbf{Q}_2}\right], \tag{4}$$

$$A_2 = \frac{8T}{N}\sum_{\mathbf{k},\omega_p}\left[(G_{\mathbf{k}})^2(G_{\mathbf{k}-\mathbf{Q}_1})^2(G_{\mathbf{k}+\mathbf{Q}_1})^2 + 3(G_{\mathbf{k}})^2(G_{\mathbf{k}-\mathbf{Q}_1})^2 G_{\mathbf{k}+\mathbf{Q}_2}G_{\mathbf{k}+\mathbf{Q}_3}\right], \tag{5}$$

$$A_3 = -\frac{4T}{N}\sum_{\mathbf{k},\omega_p}\left[(G_{\mathbf{k}})^2(G_{\mathbf{k}-\mathbf{Q}_1})^2(G_{\mathbf{k}+\mathbf{Q}_1})^2 + 2(G_{\mathbf{k}})^2(G_{\mathbf{k}-\mathbf{Q}_1})^2 G_{\mathbf{k}+\mathbf{Q}_2}G_{\mathbf{k}+\mathbf{Q}_3} - 2(G_{\mathbf{k}})^2 G_{\mathbf{k}-\mathbf{Q}_1}G_{\mathbf{k}+\mathbf{Q}_1}G_{\mathbf{k}+\mathbf{Q}_2}G_{\mathbf{k}-\mathbf{Q}_3}\right]. \tag{6}$$

The effective interaction for negative $\mathbf{Q}$, $F^{(6-)}$, is obtained by taking $\pm\mathbf{Q}_\nu \to \mp\mathbf{Q}_\nu$ in $F^{(6+)}$.

By using the identity relation as

$$[\mathbf{S}_{\mathbf{Q}_1}\cdot(\mathbf{S}_{\mathbf{Q}_2}\times\mathbf{S}_{\mathbf{Q}_3})]^2 = (\mathbf{S}_{\mathbf{Q}_1}\cdot\mathbf{S}_{\mathbf{Q}_1})(\mathbf{S}_{\mathbf{Q}_2}\cdot\mathbf{S}_{\mathbf{Q}_2})(\mathbf{S}_{\mathbf{Q}_3}\cdot\mathbf{S}_{\mathbf{Q}_3}) + 2(\mathbf{S}_{\mathbf{Q}_1}\cdot\mathbf{S}_{\mathbf{Q}_2})(\mathbf{S}_{\mathbf{Q}_2}\cdot\mathbf{S}_{\mathbf{Q}_3})(\mathbf{S}_{\mathbf{Q}_3}\cdot\mathbf{S}_{\mathbf{Q}_1})$$
$$-(\mathbf{S}_{\mathbf{Q}_1}\cdot\mathbf{S}_{\mathbf{Q}_1})(\mathbf{S}_{\mathbf{Q}_2}\cdot\mathbf{S}_{\mathbf{Q}_3})^2 - (\mathbf{S}_{\mathbf{Q}_2}\cdot\mathbf{S}_{\mathbf{Q}_2})(\mathbf{S}_{\mathbf{Q}_3}\cdot\mathbf{S}_{\mathbf{Q}_1})^2 - (\mathbf{S}_{\mathbf{Q}_3}\cdot\mathbf{S}_{\mathbf{Q}_3})(\mathbf{S}_{\mathbf{Q}_1}\cdot\mathbf{S}_{\mathbf{Q}_2})^2, \tag{7}$$

one can find that the magnetic chirality interaction in equation (4) in Methods is included in the expansion of the sixth-order term of the free energy. Thus, the L term is a representative lowest-order contribution relevant to the phase shift. The derivation above indicates that the coefficient L depends on the electronic band structure of itinerant electrons through Green's functions. Although the computation is highly complicated, one can in principle evaluate L once the electronic band structure is given.

Supplementary References

[1] S. Hayami, R. Ozawa, and Y. Motome, Phys. Rev. B **95**, 224424 (2017).
